# Comparison of Intravenous Dexmedetomidine versus Esmolol for Attenuation of Hemodynamic Response to Tracheal Intubation after Rapid Sequence Induction: A Systematic Review and Meta-Analysis

**DOI:** 10.1155/2019/6791971

**Published:** 2019-04-17

**Authors:** Zhao Li, Li Xu, Jinwei Zheng, Qingxiu Wang

**Affiliations:** ^1^Department of Anesthesiology, East Hospital, Tongji University School of Medicine, Shanghai 200120, China; ^2^Department of Anesthesiology, The First People's Hospital of Changde, Changde 415000, China; ^3^Department of Anesthesiology, Ningbo No. 2 Hospital, Ningbo 315000, China

## Abstract

The present study aims to investigate whether intravenous dexmedetomidine shows superiority to esmolol for hemodynamic response to tracheal intubation after rapid sequence induction. In the present meta-analysis, PubMed, EMBASE, and the Cochrane Library were searched for trials comparing dexmedetomidine with esmolol for the attenuation of the hemodynamic response to intubation. Ten trials were selected in the present meta-analysis. Compared to esmolol, the use of dexmedetomidine maintains stable heart rates (HR), systolic blood pressure (SBP), diastolic blood pressure (DBP), and mean arterial pressure (MAP) at 1 min, 3 min, and 5 min after tracheal intubation. Dexmedetomidine causes less hemodynamic response to tracheal intubation after rapid sequence induction compared with esmolol.

## 1. Introduction

Endotracheal intubation is the most effective procedure for managing the airway before surgery. Nevertheless, both laryngoscopy and endotracheal intubation produce marked sympathetic response that give rise to tachycardia and hypertension. The fluctuation of hemodynamic values may strongly increase the incidence of myocardial ischemia and other complications to patients with cardiovascular disease [1]. To control the increase in these hemodynamic values, numerous pharmacological and physiological prophylactic interventions have been used. But those interventions might be associated with some side effects. Thus, still an ideal intervention is needed to be discovered [2].

Dexmedetomidine, a novel *α*_2_-adrenergic agonist, possesses anxiolytic, sedative, analgesic, and sympatholytic properties with minimal respiratory depression [3]. Esmolol, an ultra-short *β*-adrenergic blocking drug, possesses little sedative effect, but no analgesic activity [4]. Compared to opioid receptor agonist, the pharmacologic properties of dexmedetomidine and esmolol suggested that these drugs may be a suitable intervention for attenuating hemodynamic response to tracheal intubation without interfering with the recovery process and causing significant respiratory depression.

However, the question of whether dexmedetomidine for attenuating hemodynamic response to tracheal intubation is superior to esmolol remains unclear. Therefore, in this study, we conducted a meta-analysis to compare the effectiveness between dexmedetomidine and esmolol for attenuating hemodynamic response to tracheal intubation during general anesthesia.

## 2. Materials and Methods

### 2.1. Search Strategy

We conducted a systematic search of the literature for all relevant randomized clinical trials. PubMed, EMBASE, and the Cochrane Library were searched from inception to July 10, 2018. There was no restriction imposed on publication status. The language of publication was limited to English. The group included in searches was limited to adults only. Reference lists of included studies were evaluated by the investigators to identify additional relevant studies. Endnote X7 was used to combine and remove duplicate citations.

### 2.2. Selection Criteria

The review was conducted according to the Cochrane Handbook for Systematic Reviews of Interventions and the PRISMA (Preferred Reporting Items for Systematic Reviews and Meta-Analyses) guidelines [5].

The studies which compared the effectiveness of dexmedetomidine and esmolol on hemodynamic response to tracheal intubation were considered relevant to this meta-analysis, regardless of the type of surgical procedure. These included studies must meet the following criteria: (1) the study should have a randomized controlled trial (RCT) design; (2) randomly assigned to receive intravenous dexmedetomidine or esmolol; (3) the patients underwent surgeries with tracheal intubation; (4) the study recorded hemodynamic parameters. The studies which did not have one of the following parameters were not included: the hemodynamic parameter number at 1 minute, 3 minutes, and 5 minutes after tracheal intubation.

### 2.3. Data Extraction and Quality Assessment

Two of the evaluators independently gathered and reviewed the titles and abstracts of retrieved studies to identify those records that appeared to meet the inclusion criteria. The full text of these articles was then obtained for further independent assessment of eligibility. Any disagreement was resolved by discussion and the third author arbitrated when necessary.

Two investigators independently recorded the following data from each study: the name of the first author, publication year, patients' ASA classification, patients' age, intervention, comparison, and drug for induction of anesthesia. Again, discrepancies in data extraction were resolved by discussion and the third author arbitrated when necessary.

Two investigators independently scanned the quality of each study according to the Cochrane Collaboration [6]. The tool contains the following domains: (1) randomization and sequence generation, (2) allocation concealment, (3) blinding method, (4) incomplete outcome data, (5) selective outcome reporting, and (6) other sources of bias. The studies were rated as having a low, high, or unclear risk of bias. Discrepancies were resolved by consensus after discussion; a third reviewer participated in the debate to determine the final outcomes if necessary.

### 2.4. Outcomes Assessed

Anticipated outcomes of included RCTs included heart rates (HR), Systolic Blood Pressure (SBP), diastolic blood pressure (DBP), and mean arterial blood pressure (MAP) measured at one, three, and five minutes after intubation.

### 2.5. Statistical Analysis

Statistical analysis was performed using the Review Manager 5.3 software (the Nordic Cochrane Centre, Copenhagen, Denmark). The pooled differences of the hemodynamic to tracheal intubation were expressed as mean difference (MD) with 95% confidence intervals (CIs). Meta-analysis was not performed if the study did not exhibit standardized mean difference or standard error of the mean (SEM).

Continuous data were analyzed by using random effect models and the risk ratio (RR) was computed using the Mantel-Haenszel method (fixed or random models). I-square (I^2^) test was performed to assess the impact of study heterogeneity on the results of the meta-analysis. According to the Cochrane review guidelines, if severe heterogeneity was present at I^2^ >50%, the random effect models were chosen; otherwise the fixed effect models were used. Differences with a p-value of <0.05 were considered statistically significant unless where otherwise specified. Given that the number of included trials in this meta-analysis was not more than 10, the publication bias was not evaluated.

## 3. Results

**Figure 1:**
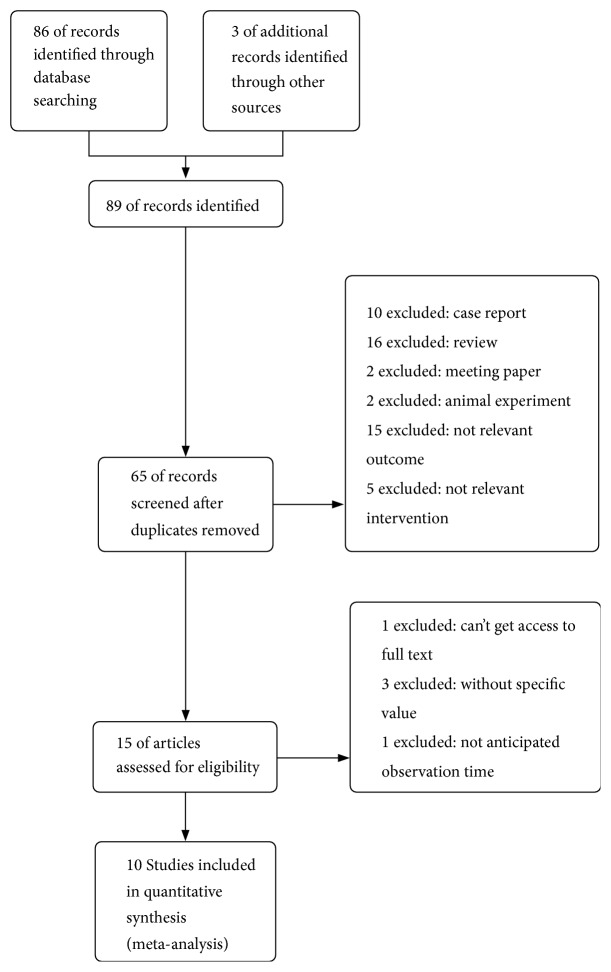
The flow chart of trial selection in the meta-analysis.

A total of 89 records were identified through database searching. After excluding duplicate studies, we obtained 65 articles of the remaining studies for further assessment. In the end, 10 RCT studies were identified in this meta-analysis [7–16]. The flowchart of the literature search and selection is shown in Figure 1. Neither bradycardia nor hypotension was reported in the reviewed trails.

### 3.1. Study Characteristics and Quality Assessment

The characteristics of the included studies are shown in Table 1. Our analysis focused only on a comparison of intervention: dexmedetomidine compared with esmolol.

**Figure 2:**
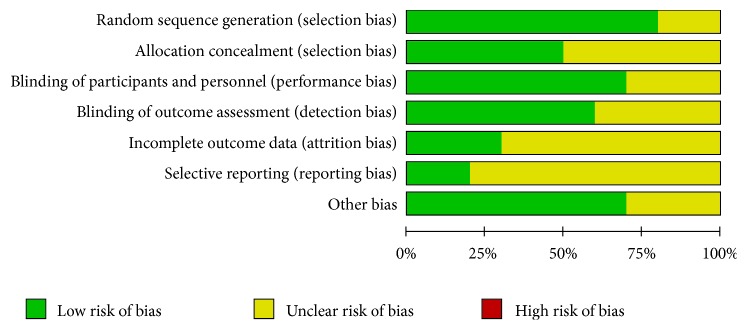
The risk of bias assessment of the included trials.

**Figure 3:**
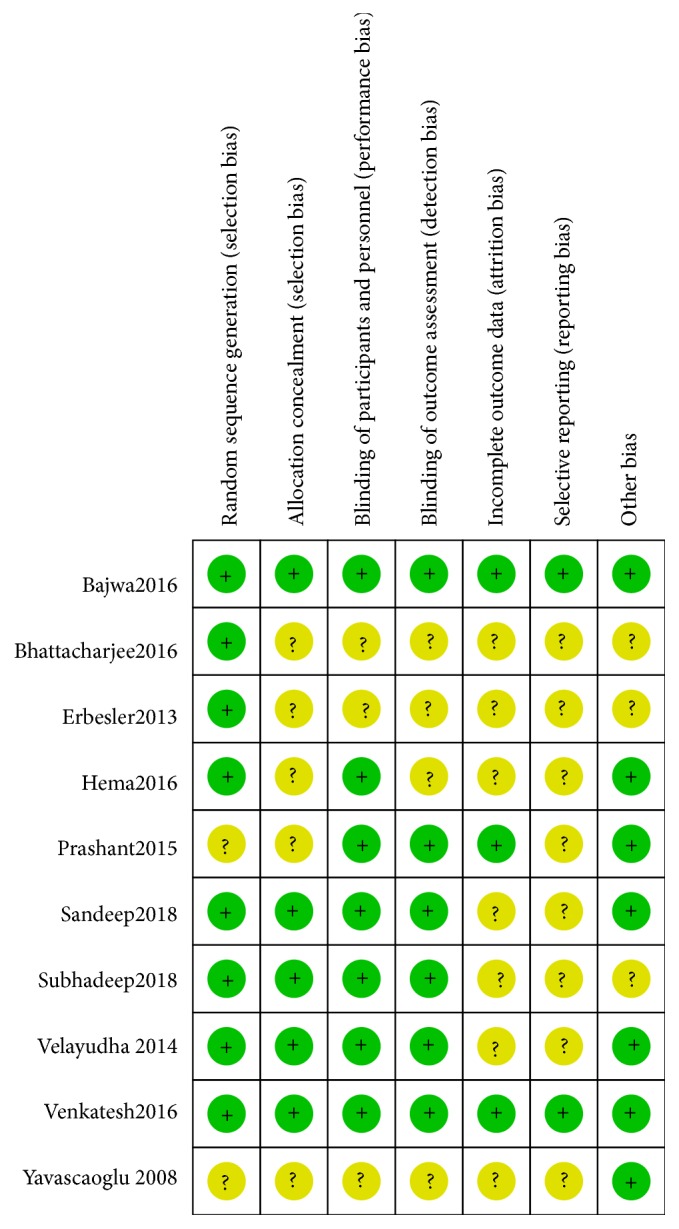
The risk of bias summary of the included trials.

Risk of bias of all the RCTs included in our meta-analysis is shown in Figures 2 and 3. Overall, the included RCTs suggested good quality in terms of risk of bias.

### 3.2. Meta-Analysis Results

**Figure 4:**
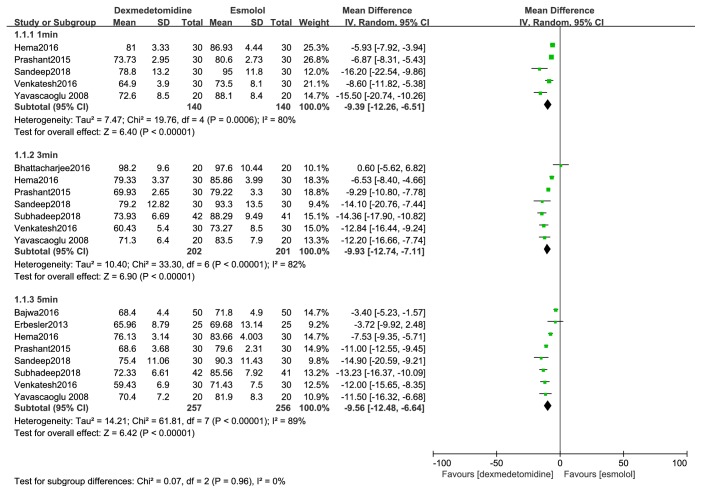
Forest plot of the heart rate for hemodynamic response to tracheal intubation using the dexmedetomidine compared with esmolol.

HR compared with esmolol: dexmedetomidine attenuated the rise of heart rate at one, three, and five minutes after tracheal intubation (MD, -9 beats/min; 95% CI, -12 to -7; MD, -10 beats/min; 95% CI, -13 to -7; MD, -10 beats/min; 95% CI, -12 to -7) (Figure 4).

**Figure 5:**
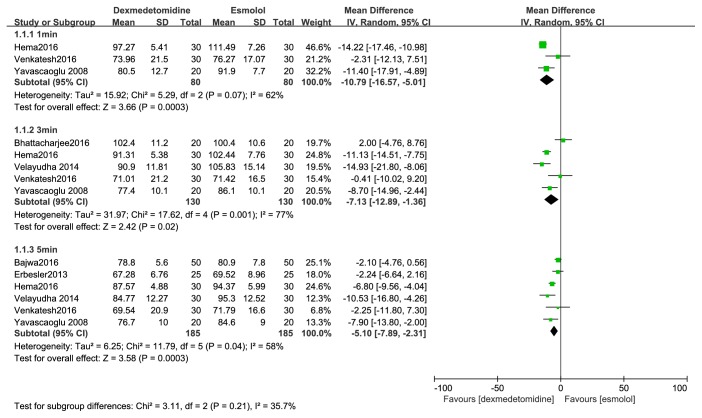
Forest plot of the mean arterial pressure for hemodynamic response to tracheal intubation using the dexmedetomidine compared with esmolol.

MAP compared with esmolol: dexmedetomidine attenuated the rise of mean arterial pressure at one, three, and five minutes after tracheal intubation (MD, -11 mmHg; 95% CI, -17 to -5; MD, -7 mmHg; 95% CI, -13 to -1; MD, -5 mmHg; 95% CI, -8 to -2) (Figure 5).

**Figure 6:**
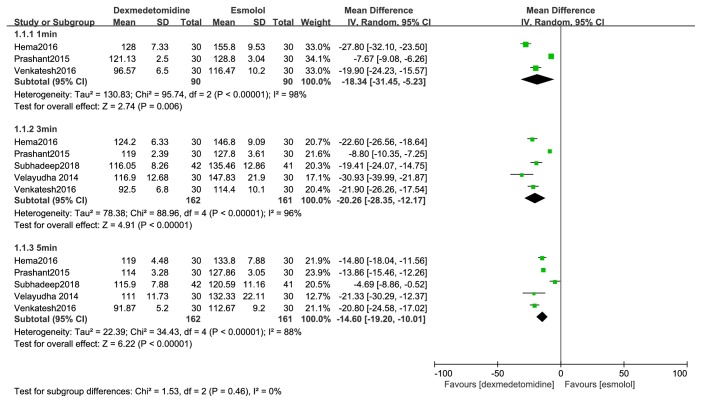
Forest plot of the systolic blood pressure for hemodynamic response to tracheal intubation using the dexmedetomidine compared with esmolol.

SBP compared with esmolol: dexmedetomidine attenuated the rise of systolic blood pressure at one, three, and five minutes after tracheal intubation (MD, -18 mmHg; 95% CI, -31 to -5; MD, -20 mmHg; 95% CI, -28 to -12; MD, -15 mmHg; 95% CI, -19 to -10) (Figure 6).

**Figure 7:**
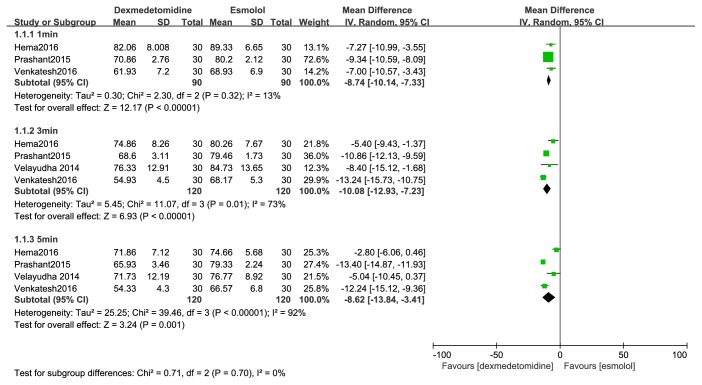
Forest plot of the diastolic blood pressure for hemodynamic response to tracheal intubation using the dexmedetomidine compared with esmolol.

DBP compared with esmolol: dexmedetomidine attenuated the rise of diastolic blood pressure at one, three, and five minutes after tracheal intubation (MD, -9 mmHg; 95% CI, -10 to -7; MD, -10 mmHg; 95% CI, -13 to -7; MD, -9 mmHg; 95% CI, -14 to -3) (Figure 7).

## 4. Discussion

This meta-analysis revealed that, compared to esmolol, dexmedetomidine is a more effective agent for attenuating the hemodynamic response to tracheal intubation during general anesthesia. Dexmedetomidine had better control over heart rate and blood pressure. Therefore, compared to esmolol, dexmedetomidine is a more effective intervention to reduce the hemodynamic response to tracheal intubation.

The hemodynamic response to laryngoscopy and endotracheal intubation involves a pressor response, which leads to transient hypertension and tachycardia [17]. Though the increases in HR and BP may be tolerant in healthy patients, they may be potentially hazardous to those with cardiovascular and cerebrovascular diseases [18].

Plenty of interventions have been used to attenuate the pressor response including inhalation anesthetics, local anesthetics, calcium channel blockers, opioid analgesics, and vasodilators, but they have got some side effects such as hypotension, bradycardia, and respiratory depression.

There is entirely different pharmacokinetic profile between dexmedetomidine and esmolol. The transient hypertension and tachycardia are caused by the secretion of adrenaline and noradrenaline. But adrenaline is only centrally secreted and part of noradrenaline is peripherally secreted [19]. Esmolol is a water-soluble agent, which cannot pass through the blood-brain barrier and does not likely play a potential role in the central *β* receptor [20].

The attenuation of hypertension and tachycardia by esmolol may cover the unstable status. Meanwhile, the increase of peripheral resistance and cardiac load may aggravate the risk of myocardial ischemia, especially for the patients with cardiovascular and cerebrovascular diseases. In contrast, dexmedetomidine is a central alpha-2 agonist, which attenuates stress response, thus creating a more stable hemodynamic effect [21].

This meta-analysis has the following limitations: The studies included were mainly in Middle East and Asia, so whether the conclusion may be suitable to other populations including American and European is unclear. The studies included in this meta-analysis only evaluated normotensive patients. The outcome may not be applied to the hypertensive patients who require more stable intubation response. Further studies should be focused on any adverse effect associated with the use of dexmedetomidine and esmolol in the perioperative period.

In all, compared to esmolol, dexmedetomidine blunts the hemodynamic response to tracheal intubation after rapid sequence induction.

## 5. Conclusions

The results of the present meta-analysis revealed that dexmedetomidine had better control over heart rate and blood pressure; compared to esmolol, dexmedetomidine has a more clinical advantage in the perioperative period.

## Figures and Tables

**Table 1:** Baseline characteristics of included studies.

Study name	Year	ASA classification	Age (yr)	Intervention	Comparison	Drug for induction of anesthesia
Subhadeep Chakraborty	2018	I and II	30-60	dexmedetomidine 1 *μ*g/kg	esmolol 2 mg/kg	ondansetron 0.08 mg/kg, glycopyrrolate 0.004 mg/kg, midazolam 0.02 mg/kg, thiopentone sodium 5 mg/kg and intubated after succinylcholine 2 mg/kg

Sandeep Sharma	2018	I and II	20-50	dexmedetomidine 1 *μ*g/kg	esmolol 1.5 mg/kg	midazolam 0.03 mg/kg, fentanyl 1 *μ*g/kg, propofol 2.5 mg/kg and succinylcholine 2 mg/kg

Venkatesh Selvaraj	2016	I	18–60	dexmedetomidine 1 *μ*g/kg	esmolol 0.5 mg/kg	NR

Hema B	2016	I and II	18–60	dexmedetomidine 1 *μ*g/kg	esmolol 1 mg/kg	glycopyrrolate 0.004 mg/kg, fentanyl 1*μ*g/kg, thiopentone 5 mg/kg and vecuronium 0.1mg/kg

Bajwa S. J.	2016	I and II	18–55	dexmedetomidine 1 *μ*g/kg	esmolol 1 mg/kg	thiopentone 5 mg/kg, fentanyl 1*μ*g/kg and vecuronium 0.1mg/kg

Bhattacharjee D. P.	2016	I and II	20-60	dexmedetomidine 1 *μ*g/kg	esmolol 0.5 mg/kg	fentanyl 2 *μ*g/kg, propofol 2 mg/kg and rocuronium 0.7 mg/kg

Prashant Dass	2015	I and II	18–65	dexmedetomidine 0.5 *μ*g/kg	esmolol 0.3 mg/kg	thiopental sodium 2 *μ*g/kg, fentanyl 2 *μ*g/kg and vecuronium bromide 0.1 mg/kg

Velayudha Reddy	2014	I and II	40-60	dexmedetomidine 1 *μ*g/kg	esmolol 2 mg/kg	ondansetron 0.08 mg/kg, glycopyrrolate 0.004 mg/kg, thiopentone 5.0 mg/kg and succinylcholine 2.0 mg/kg

Erbesler Z. A.	2013	I and II	18–65	dexmedetomidine 0.5 *μ*g/kg	esmolol 0.5 mg/kg	0.03 mg midazolam, propofol 2 mg/kg, remifentanil 1 *μ*g/kg and rocuronium 0.6 mg/kg

B. Yavascaoglu	2008	I and II	18-60	dexmedetomidine 0.5 *μ*g/kg	esmolol 0.3 mg/kg	midazolam 0.03mg kg, fentanyl 2 *μ*g/kg, rocuronium 0.6 mg/kg and propofol NR

ASA: American Society of Anesthesiologists-Physical Status; yr: years; NR: not recorded.
